# Antibacterial Characterization of Novel Synthetic Thiazole Compounds against Methicillin-Resistant *Staphylococcus pseudintermedius*

**DOI:** 10.1371/journal.pone.0130385

**Published:** 2015-06-18

**Authors:** Haroon Mohammad, P. V. Narasimha Reddy, Dennis Monteleone, Abdelrahman S. Mayhoub, Mark Cushman, G. Kenitra Hammac, Mohamed N. Seleem

**Affiliations:** 1 Department of Comparative Pathobiology, Purdue University College of Veterinary Medicine, West Lafayette, Indiana, United States of America; 2 Department of Medicinal Chemistry and Molecular Pharmacology, Purdue University College of Pharmacy West Lafayette, Indiana, United States of America; 3 Purdue Center for Cancer Research, West Lafayette, Indiana, United States of America; The University of Hong Kong, HONG KONG

## Abstract

*Staphylococcus pseudintermedius* is a commensal organism of companion animals that is a significant source of opportunistic infections in dogs. With the emergence of clinical isolates of *S*. *pseudintermedius* (chiefly methicillin-resistant *S*. *pseudintermedius* (MRSP)) exhibiting increased resistance to nearly all antibiotic classes, new antimicrobials and therapeutic strategies are urgently needed. Thiazole compounds have been previously shown to possess potent antibacterial activity against multidrug-resistant strains of *Staphylococcus aureus* of human and animal concern. Given the genetic similarity between *S*. *aureus* and *S*. *pseudintermedius*, this study explores the potential use of thiazole compounds as novel antibacterial agents against methicillin-sensitive *S*. *pseudintermedius* (MSSP) and MRSP. A broth microdilution assay confirmed these compounds exhibit potent bactericidal activity (at sub-microgram/mL concentrations) against both MSSA and MRSP clinical isolates while the MTS assay confirmed three compounds (at 10 μg/mL) were not toxic to mammalian cells. A time-kill assay revealed two derivatives rapidly kill MRSP within two hours. However, this rapid bactericidal activity was not due to disruption of the bacterial cell membrane indicating an alternative mechanism of action for these compounds against MRSP. A multi-step resistance selection analysis revealed compounds **4** and **5** exhibited a modest (two-fold) shift in activity over ten passages. Furthermore, all six compounds (at a subinihibitory concentration) demonstrated the ability to re-sensitize MRSP to oxacillin, indicating these compounds have potential use for extending the therapeutic utility of β-lactam antibiotics against MRSP. Metabolic stability analysis with dog liver microsomes revealed compound **3** exhibited an improved physicochemical profile compared to the lead compound. In addition to this, all six thiazole compounds possessed a long post-antibiotic effect (at least 8 hours) against MRSP. Collectively the present study demonstrates these synthetic thiazole compounds possess potent antibacterial activity against both MSSP and MRSP and warrant further investigation into their use as novel antimicrobial agents.

## Introduction

*Staphylococcus pseudintermedius* is a significant problem in veterinary medicine as it is a major source of opportunistic infections in companion animals and the leading causative agent of canine pyoderma [1]. It has also been linked to other severe infections in companion animals including urinary tract infections, skin wounds, surgical site infections, and otitis [2–4]. The challenge to combat *S*. *pseudintermedius* infections has become more difficult with the emergence of clinical isolates (primarily methicillin-resistant *S*. *pseudintermedius*) exhibiting resistance to multiple antibiotic classes including β-lactams, lincosamides, fluoroquinolones, macrolides, sulfonamides, aminoglycosides, tetracyclines, and chloramphenicol [1, 5, 6]. In several cases, patients that contracted an infection caused by *S*. *pseudintermedius*, in particular newborn puppies, have died or been euthanized due to the lack of effective treatment to remedy the medical condition [4, 7, 8]. Thus there is a critical need for the discovery and characterization of novel antimicrobials to treat infections caused by methicillin-sensitive and methicillin-resistant *S*. *pseudintermedius*.

Thiazole compounds have been shown to be useful in multiple therapeutic applications including as anticancer, antiviral, and anticonvulsant agents [9–11] but their potential use as antibacterials has not been fully examined. Previous investigation into thiazole compounds synthesized by our research group has revealed these compounds exhibit potent antimicrobial activity against multidrug-resistant strains of *Staphylococcus aureus*, including methicillin-resistant *S*. *aureus* (MRSA) [12, 13]. MRSP bears similar genetic and phenotypic traits to MRSA, including expression of the *mecA* gene that encodes a modified penicillin-binding protein that confers resistance to β-lactam antibiotics [14]. Additionally, *S*. *pseudintermedius* has been shown to express surface proteins similar to *S*. *aureus* that play an important role in bacterial colonization of host tissues [15]. Furthermore, both staphylococcal species secrete similar virulence factors, including exfoliative toxins and leukocidins, that may play an important role in promoting pathogenesis of disease in infected hosts [16–18].

**Fig 1:**
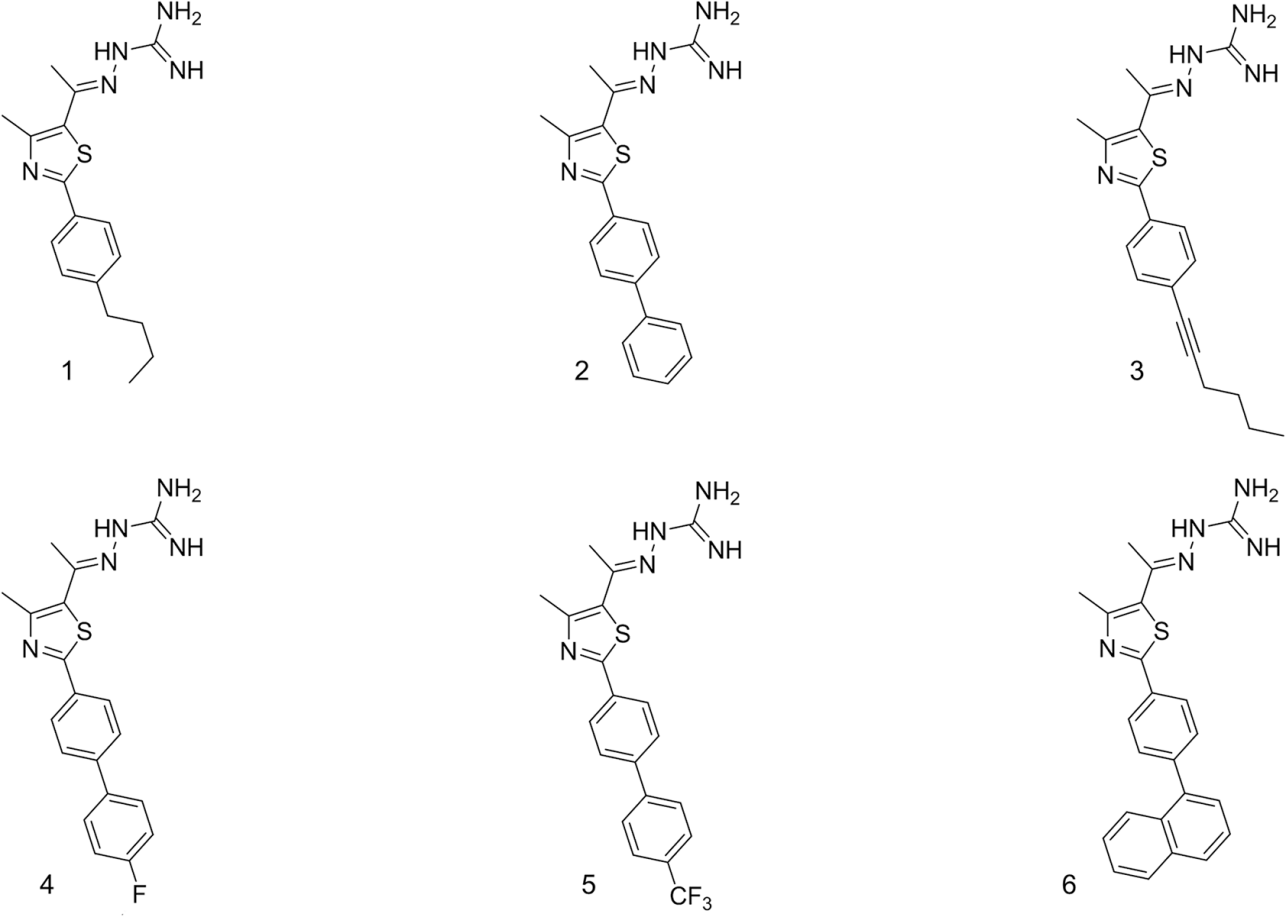
Chemical structures of thiazole compounds 1–6 utilized in this study.

Given the genetic and phenotypic similarities between *S*. *pseudintermedius* and *S*. *aureus*, we suspected that the thiazole compounds we have found to be potent inhibitors of MRSA would also be active against MRSP. The objectives of the present study were to characterize the antibacterial activity of six of the most potent thiazole compounds (against MRSA) (Fig 1) against clinical isolates of MSSP and MRSP, to ascertain the likelihood of MRSP acquiring rapid resistance to these novel compounds, and to determine if the compounds could be used to re-sensitize MRSP to the effect of β-lactam antibiotics. Additionally, we assessed the physicochemical profile of the most promising analogue and examined the ability of MRSP to recover after exposure to the thiazole antibiotics, via a post-antibiotic effect assay. The results garnered lend valuable insight into the pharmacological utility of thiazole compounds as a possible future therapeutic option for the treatment of *S*. *pseudintermedius* infections.

## Materials and Methods

### Bacterial isolates and chemical reagents

Fifteen isolates of *S*. *pseudintermedius* (nine MSSP and six MRSP), identified at the Indiana Animal Disease Diagnostic Laboratory from specimens collected from dogs admitted to the small animal teaching hospital at Purdue University, were included in the study. The specimens were not collected specifically for this research study but were obtained from patients admitted to the hospital for treatment. Clinical specimens were inoculated onto 5% sheep blood agar and incubated at 35°C for 18–24 hours. Standard methods including examination of colony morphology and hemolysis and biochemical tests such as tube coagulase, Voges-Proskauer (VP) and fermentation tests for maltose, trehalose and lactose, were used in addition to matrix-assisted laser desorption ionization time of flight mass spectrometry [19] to identify isolates [19, 20]. Antimicrobial susceptibility was determined by broth microdilution using the SensiTitre (Thermofisher Scientific). Isolates demonstrating resistance to oxacillin, a surrogate for methicillin, with a minimum inhibitory concentration (MIC) value greater than or equal to 0.5 μg/mL were tested for the presence of *mecA* by PCR as previously described [21]. Bacterial isolates used in this study are presented in Table 1.

**Table 1:** Clinical isolates of *Staphylococcus pseudintermedius* used in this study.

Methicillin-sensitive *Staphylococcus pseudintermedius* isolates					
Isolate Name	Species	Breed	Age	Origin	Resistance Phenotype^a^
SP1	Canine	Mixed	9 years	Urine	PEN, AMP
SP2	Canine	Cocker Spaniel	10.5 years	Urine	PEN, AMP, CLIN, ENRO, ERYTH, GEN, MARBO, TMP-SMX
SP6	Canine	Labrador Retriever	6 years	Ear	None
SP7	Canine	Cocker Spaniel	10 years	Ear	AMK, PEN, AMP, CLIN, ENR, ERM, GEN, MARB, TMP-SMX
SP11	Canine	Mixed	9.5 years	Ear	PEN, AMP, CHL, CLIN
SP12	Canine	West Highland White	15.5 years	Urine	PEN, AMP, AMK, ENR, GEN, MARB, TMP-SMX
SP14	Canine	Golden Retriever	4.5 years	Ear	PEN, CHL, CLIN, ERM
SP15	Canine	Mixed	9.5 years	Urine	None
SP23	Canine	Boxer	9.5 years	Wound	PEN, AMP
**Methicillin-resistant *Staphylococcus pseudintermedius* isolates**					
SP3	Canine	English Bulldog	8 months	Orthopedic implant	AMP, PEN, AMO, CEF, ERM, CLIN, IMI,OXA, TIC
SP5	Canine	Mixed	10.5 years	Urine	AMK, AMP, PEN, AMO, CEF, CHL, CLIN, ENR, ERM, GEN, IMI, MARB, OXA, TIC, TMP-SMX
SP8	Canine	Maltese	10 years	Urine	AMP, PEN, AMO, CEF, ERM, CLIN, IMI, OXA, TIC, CHL
SP9	Canine	Mixed	4 years	Skin	AMK, AMP, PEN, AMO, CEF, CHL, CLIN, ENR, ERM, GEN, IMI, MARB, OXA, TIC, TMP-SMX
SP25	Canine	Mixed	11.5 years	Urine	AMK, AMP, PEN, AMO, CEF, CHL, CLIN, ENR, ERM, GEN, IMI, MARB, OXA, TIC, TMP-SMX
SP28	Canine	West Highland White	15 years	Urine	AMK, AMP, PEN, AMO, CEF, CHL, CLIN, ENR, ERM, GEN, IMI, MARB, OXA, TIC, TMP-SMX

^a^Abbreviations: PEN: penicillin, AMP, ampicillin, AMK: amikacin, CEF: cefpodoxime, CLIN: clindamycin, GEN: gentamycin, CHL: chloramphenicol, ENR: enrofloxacin, MARB: marbofloxacin, ERM: erythromycin, TMP-SMX: trimethoprim/sulfamethoxazole, TIC: ticarcillin, IMI: imipenem, AMO: amoxicillin, OXA: oxacillin.

Clindamycin hydrochloride monohydrate (Tokyo Chemical Industry, Portland, OR, USA), oxacillin sodium salt monohydrate (Tokyo Chemical Industry, Portland, OR, USA), rifampicin (Sigma-Aldrich, St. Louis, MO, USA), and vancomycin hydrochloride (Gold Biotechnology, St. Louis, MO, USA) were purchased commercially. All antibiotics were dissolved in dimethyl sulfoxide (DMSO) to obtain a stock 10 mM solution.

### Synthesis of thiazole compounds 1–6

The detailed synthetic protocols and spectral data of final products **1**–**6** as well as all intermediates have been previously reported [12, 22, 23]. Chemical structures of compounds **1**–**6** are presented in Fig 1. All compounds were dissolved in DMSO to obtain a stock 10 mM solution.

### Determination of minimum inhibitory concentration and minimum bactericidal concentration (MBC) against S. pseudintermedius

The MICs of the thiazole compounds, clindamycin, and rifampicin against nine clinical isolates of MSSP and six clinical isolates of MRSP were determined using the broth microdilution method, in accordance with the recommendations contained in the CLSI guidelines [24]. Bacteria were prepared in phosphate-buffered saline (PBS) until a McFarland standard of 0.5 was achieved. The solution was subsequently diluted 1:300 in Mueller-Hinton broth (MHB) to reach a starting inoculum of 1 × 10^5^ colony-forming units (CFU/mL). Bacteria were then transferred to a 96-well microtiter plate. Thiazole compounds and antibiotics were added (in triplicate) to wells in the first row of the microtiter plate and then serially diluted along the vertical axis. The plate was incubated at 37°C for 22–24 hours before the MIC was determined. The MIC was categorized as the concentration where there was no visible growth of bacteria observed.

The MBC was determined by plating 5 μL from wells on the 96-well microtiter plate (where the MIC was determined) where no growth was observed onto Tryptic soy agar (TSA) plates. The TSA plates were then incubated at 37°C for 22–24 hours before the MBC was determined. The MBC was categorized as the concentration where ≥99% reduction in bacterial cell count was observed.

### Time-kill analysis of thiazole compounds and antibiotics against MRSP

MRSP SP3 cells in late logarithmic growth phase were diluted to ~1 × 10^6^ CFU/mL and exposed to concentrations equivalent to 4 × MIC (in triplicate) of thiazole compounds **1**–**6** and rifampicin in MHB. Samples (20 μL) were collected after 0, 2, 4, 6, 8, 10, 12, and 24 hours of incubation at 37°C and subsequently serially diluted in PBS. Bacteria were then transferred to TSA plates and incubated at 37°C for 20–24 hours before viable CFU/mL values were determined.

### Cell membrane disruption analysis

In order to investigate the antimicrobial effect of the thiazole compounds on the integrity of the bacterial cell membrane, the release of 260 and 280 nm absorbing components was determined spectrophotometrically [25]. The cell suspension of 1.2 × 10^9^ CFU/mL MRSP SP3 was incubated with 4 × MIC of compound **2** at 37°C for 30 minutes. Untreated MRSP SP3 cells or cells treated with vancomycin (inhibits cell wall synthesis in bacterial cells) served as negative controls. For the release of 260 and 280 nm absorbing material, the bacterial suspension (control) was treated with lysostaphin (in 50 mM Tris-HCl, pH 8.00) for 30 minutes. Lysostaphin was used as a positive control due to its mode of action being the disruption of the cross-linking of the pentaglycin bridges in the cell wall of staphylococci bacteria [26]. The absorbance of cell supernatant at 260 and 280 nm was determined using a spectrophotometer (Jenway 6305). The average OD_260_ and OD_280_ values of duplicates of each treatment option were calculated and expressed as the proportion of average OD_260_ (or OD_280_) for each treatment option compared to the average OD_260_ (or OD_280_) for the positive control (lysostaphin).

### *In vitro* cytotoxicity analysis

Compounds were assayed at concentrations of 5 μg/mL, 10 μg/mL, 20 μg/mL, and 40 μg/mL against a murine macrophage cell line (J774.A1) (ATCC TIB-67, American Type Culture Collection (ATCC), Manassas, VA, USA) to determine the potential toxic effect *in vitro*. Cells were cultured in Dulbeco’s modified Eagle’s medium (Sigma-Aldrich, St. Louis, MO, USA) with 10% fetal bovine serum (USA Scientific, Inc.) at 37°C with 5% CO_2_. Controls received DMSO alone at a concentration equal to that in drug-treated cell samples. The cells were incubated with the compounds in a 96-well plate at 37°C and 5.0% CO_2_ for two hours prior to addition of the assay reagent MTS 3-(4,5-dimethylthiazol-2-yl)-5-(3-carboxymethoxyphenyl)-2-(4-sulfophenyl)-2*H*-tetrazolium) (Promega, Madison, WI, USA). Absorbance readings were taken using a kinetic ELISA microplate reader (Molecular Devices, Sunnyvale, CA, USA). The quantity of viable cells after treatment with each compound was expressed as a percentage of the control, DMSO.

### Multi-step resistance selection

To assess the ability of MRSP to develop resistance to the thiazole compounds, a multi-step resistance selection experiment was performed, as described elsewhere [27]. The broth microdilution method for MIC determination against a clinical isolate of MRSP (SP3) was repeated for ten passages over a period of ten days. The initial inoculum was prepared to a McFarland standard of 0.5. The solution was subsequently diluted 1:300 in MHB to reach a starting inoculum of 1 × 10^5^ CFU/mL. For each subsequent passage, the inoculum for the MIC determination was adjusted to a final density of approximately 5 × 10^5^ CFU/mL using the contents of a well containing a subinhibitory concentration of the compound (where bacterial growth was observed from the previous passage). Bacteria were then transferred to a new 96-well microtiter plate. Thiazole compounds **3–6** and clindamycin were added (in triplicate) to wells in the first row of the microtiter plate and then serially diluted along the ordinate. The plate was incubated at 37°C for 22 hours before the MIC was determined by visual inspection. Resistance was classified as a greater than four-fold increase in the initial MIC, as reported elsewhere [28].

### Combination therapy assessment of thiazole compounds with oxacillin

The relationship between the thiazole compounds and oxacillin was assessed via a standard checkerboard assay [29]. Bacteria (MRSP SP3) equivalent to a McFarland standard of 0.5 were prepared in PBS. The bacteria were next diluted in MHB to achieve a starting cell density of 1 × 10^5^ CFU/mL. MHB was transferred to all wells of a 96-well microtiter plate. The thiazole compounds and oxacillin were diluted in MHB to achieve a starting concentration equivalent to 2 × or 4 × MIC, respectively. Oxacillin was serially diluted along the abscissa of the microtiter plate while the thiazole compound was serially diluted along the ordinate. The plate was incubated for 22–24 hours at 37°C. The MIC of the test compound in combination with oxacillin was determined as the lowest concentration of each compound/antibiotic where no visible growth of bacteria was observed. The fractional inhibitory concentration index (ƩFIC) was calculated for each combination as described previously [13]. A synergistic relationship was classified as an FIC index less than or equal to 0.5. FIC values above 0.5 but less than 4.0 were characterized as indifference while FIC values above 4.0 were classified as antagonistic.

### Re-sensitization of MRSP to oxacillin using broth microdilution method

MHB was inoculated with MRSP SP3 (5×10^5^ CFU/mL), as has been previously described [30]. Aliquots (5 mL) of the bacterial suspension were divided into microcentrifuge tubes. The thiazole compounds tested (at ½ × MIC) were introduced into each tube. After sitting at room temperature for 30 minutes, 1 mL samples from each tube were transferred to a new centrifuge tube prior to addition of oxacillin (at a concentration equivalent to its MIC). Using a 96-well microtiter plate, rows 2–12 were filled with the remaining 4 mL bacterial suspension (containing the thiazole compound). Aliquots (200 μL) from tubes containing both the thiazole compound and oxacillin were transferred to row 1 of the 96-well plate. After aspirating contents in the first row 4–6 times, 100 μL was transferred from wells in row 1 to row 2. This process was repeated to dilute the remaining wells containing no antibiotic. Untreated bacteria served as a control. The plate was incubated at 37°C for 22 hours before the MIC was recorded. The MIC was categorized as the concentration at which no visible growth of bacteria was observed in a particular well. A fold reduction was calculated by comparing the MIC of the antibiotic alone compared to the MIC of the antibiotic given in combination with the thiazole compounds.

### Kinetic solubility determination of compound 3

Serial dilutions of compound **3** were prepared in DMSO at 100× the final concentration. Compound **3** was then diluted 100-fold into PBS in a 96-well plate and mixed. The absorbance of the PBS-containing plate was measured prior to addition of the test agents to determine the background absorbance. After 2 hours, the presence of precipitate was detected by turbidity (absorbance at 540 nm). An absorbance value of greater than (mean + 3× standard deviation of the blank), after subtracting the pre-experiment background, was indicative of turbidity. The solubility limit is reported as the highest experimental concentration for compound **3** with no evidence of turbidity as described previously [12].

### Microsomal stability analysis

Compound **3** was incubated in duplicate with dog liver microsomes at 37°C. The reaction contained microsomal protein in 100 mM potassium phosphate, 2 mM NADPH, 3 mM MgCl_2_, pH 7.4. A control was run for each test agent omitting NADPH to detect NADPH-free degradation. At 0, 10, 20, 40, and 60 minutes, an aliquot was removed from each experimental and control reaction and mixed with an equal volume of ice-cold Stop Solution (methanol containing haloperidol, diclofenac, or other internal standard). Stopped reactions were incubated at least 10 minutes at -20°C, and an additional volume of water was subsequently added. The samples were centrifuged to remove precipitated protein, and the supernatants were analyzed by LC/MS/MS to quantitate the remaining parent. Data were converted to % remaining by dividing by the time zero concentration value. Data were then fitted to a first-order decay model to determine half-life. Intrinsic clearance was calculated from the half-life and the protein concentrations, as has been described elsewhere [12].

### Post-antibiotic effect

To assess if the thiazole compounds exhibit a post-antibiotic effect (PAE) against MRSP, MRSP SP3 cells in late logarithmic growth phase (~1×10^8^ CFU/mL) were incubated with 4 × MIC of thiazole compounds **1**–**6**, clindamycin, or rifampicin for one hour at 37°C. A tube containing untreated bacterial cells served as a control. Afterward, the compound/antibiotic was washed out by diluting bacteria 1:1000 in MHB. Counts of CFU for all cultures were obtained after washing. Aliquots (100 μL) of bacteria were removed every hour (for 10 hours), serially diluted in PBS, and plated on TSA plates. TSA plates were incubated for 20 hours at 37°C before CFU values were determined. The PAE was calculated using the same formula described elsewhere [31]: PAE = *T* − *C*, where *T* is the time required for the count of CFU values in the test culture to increase one log_10_ above the count observed immediately after removal of the test agent and *C* is the time required for the count of CFU in the untreated control culture to increase one log_10_ above the count observed immediately after completion of the same procedure used on the test culture for removal of test agent.

### Statistical analysis

All statistical analyses were performed with GraphPad Prism 6.0 (GraphPad Software, La Jolla, CA). Data generated from cytotoxicity analysis of the thiazole compounds against J774.A1 cells and the 260 and 280 nm cell leakage analysis were analyzed using one-way ANOVA, with post hoc Tukey's multiple comparisons test (*P* < 0.05).

## Results

### MICs and MBCs of thiazole compounds and antibiotics against *S. pseudintermedius*

All six thiazole compounds exhibited potent antimicrobial activity against all *S*. *pseudintermedius* isolates tested (Table 2). The MIC_50_ values obtained for the compounds against methicillin-sensitive *S*. *pseudintermedius* were in close proximity to one another ranging from 0.30 μg/mL for compound **2** to 0.80 μg/mL for compound **6**. These values mimicked the results obtained for clindamycin (MIC_50_ of 0.48 μg/mL), a first-line antibiotic recommended for use in the treatment of pyoderma infections [32]. The MBC_50_ values matched or were up to three-fold higher than the MIC_50_ values determined for the thiazole compounds; this indicates that these compounds exhibit bactericidal activity against methicillin-sensitive *S*. *pseudintermedius*. The compounds retained their antimicrobial activity against the isolates of methicillin-resistant *S*. *pseudintermedius* tested. Interestingly, compounds **3** and **6** showed a nearly two-fold improvement in the MIC_50_ value obtained against MRSP isolates as compared to the MSSA isolates. The thiazoles retained their bactericidal activity against MRSP isolates with MBC_50_ values ranging from 0.42 μg/mL for compound **5** to 1.47 μg/mL for compound **4**.

**Table 2:** Minimum inhibitory concentration (MIC) and minimum bactericidal concentration (MBC) of thiazole compounds 1–6, clindamycin, and rifampicin (triplicate samples) against nine methicillin-sensitive *Staphylococcus pseudintermedius* and six methicillin-resistant *Staphylococcus pseudintermedius* isolates.

	Methicillin-sensitive *Staphylococcus pseudintermedius*				Methicillin-resistant *Staphylococcus pseudintermedius*			
Compound Number/Antibiotic	MIC_50_ ^1^ (μg/mL)	MIC Range(μg/mL)	MBC_50_ ^2^(μg/mL)	MBC Range(μg/mL)	MIC_50_ (μg/mL)	MIC Range(μg/mL)	MBC_50_(μg/mL)	MBC Range (μg/mL)
**1**	0.35	0.17–1.38	0.46	0.17–2.77	0.69	0.35–1.38	0.92	0.35–1.38
**2**	0.30	0.15–0.61	0.61	0.15–2.42	0.46	0.15–0.61	0.61	0.30–0.81
**3**	0.71	0.18–0.94	0.71	0.18–1.41	0.48	0.18–1.41	0.71	0.18–1.41
**4**	0.73	0.18–2.94	1.10	0.18–2.94	1.47	0.37–1.47	1.47	0.31–2.94
**5**	0.42	0.21–1.67	0.42	0.21–6.67	0.42	0.21–1.67	0.42	0.21–3.34
**6**	0.80	0.20–1.60	1.06	0.20–3.19	0.40	0.20–1.60	0.80	0.20–1.60
Clindamycin	0.48	0.24–61.37	0.48	0.24- >61.37	0.48	0.24- >30.69	30.69	0.24- >61.37
Rifampicin	<0.41	<0.41	<0.41	<0.41	<0.41	<0.41	<0.41	<0.41

^1^MIC_50_ corresponds to the lowest concentration of each test agent that inhibited growth in 50% of bacterial isolates screened.

^2^MBC_50_ corresponds to the lowest concentration of each test agent that killed 50% of bacterial isolates screened.

### Time-kill analysis of thiazole compounds and rifampicin

**Fig 2:**
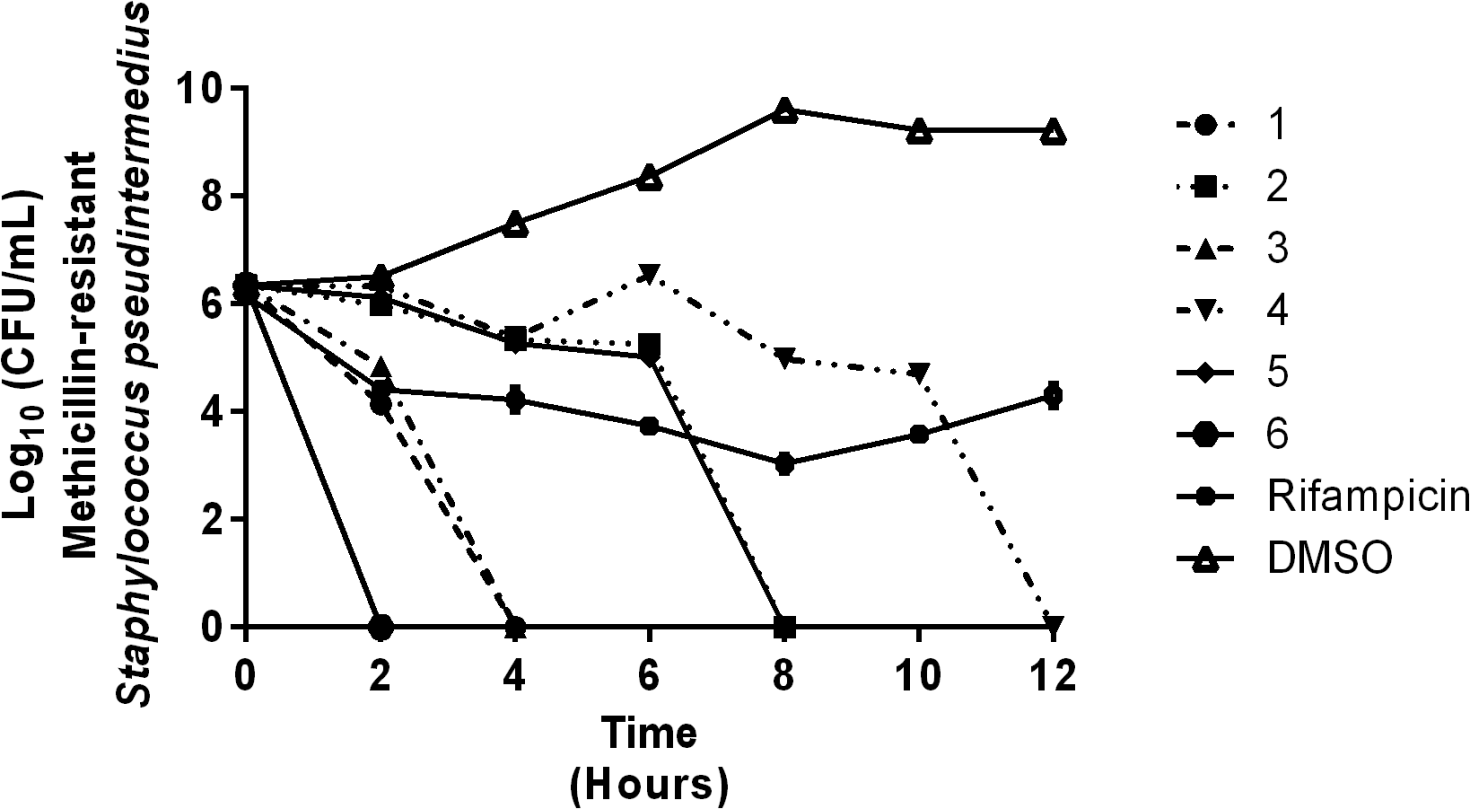
Time-kill analysis of thiazole compounds and rifampicin against methicillin-resistant *Staphylococcus pseudintermedius* SP3. Bacteria were incubated with 4 × MIC (in triplicate) of thiazole compounds or rifampicin over a 24 hour incubation period at 37°C. Samples were collected at 0, 2, 4, 6, 8, 10, 12, and 24 hours. DMSO served as a control. The error bars represent standard deviation values obtained from triplicate samples used for each compound studied.

To confirm the thiazole compounds were bactericidal against MRSP, a time-kill assay was performed using 4 × MIC of each compound and rifampicin. As Fig 2 demonstrates, the thiazole compounds are bactericidal but the rate of killing varies. The lead compound (**1**) required four hours to completely eliminate MRSP. Derivatives **3** and **6** showed improved killing kinetics, completely eliminating MRSP within two hours. Compounds **2** and **5** require eight hours to achieve the same result while compound **4** exhibits the slowest rate of bacterial killing, requiring 12 hours to completely eliminate MRSP. Rifampicin exhibited a slow bactericidal activity against MRSP SP3 within the first eight hours of the assay before rapid bacterial re-growth is observed after eight hours; this pattern is similar to what has been observed with *S*. *aureus* in other studies [33–35].

### MRSP cell membrane disruption assessment

**Fig 3:**
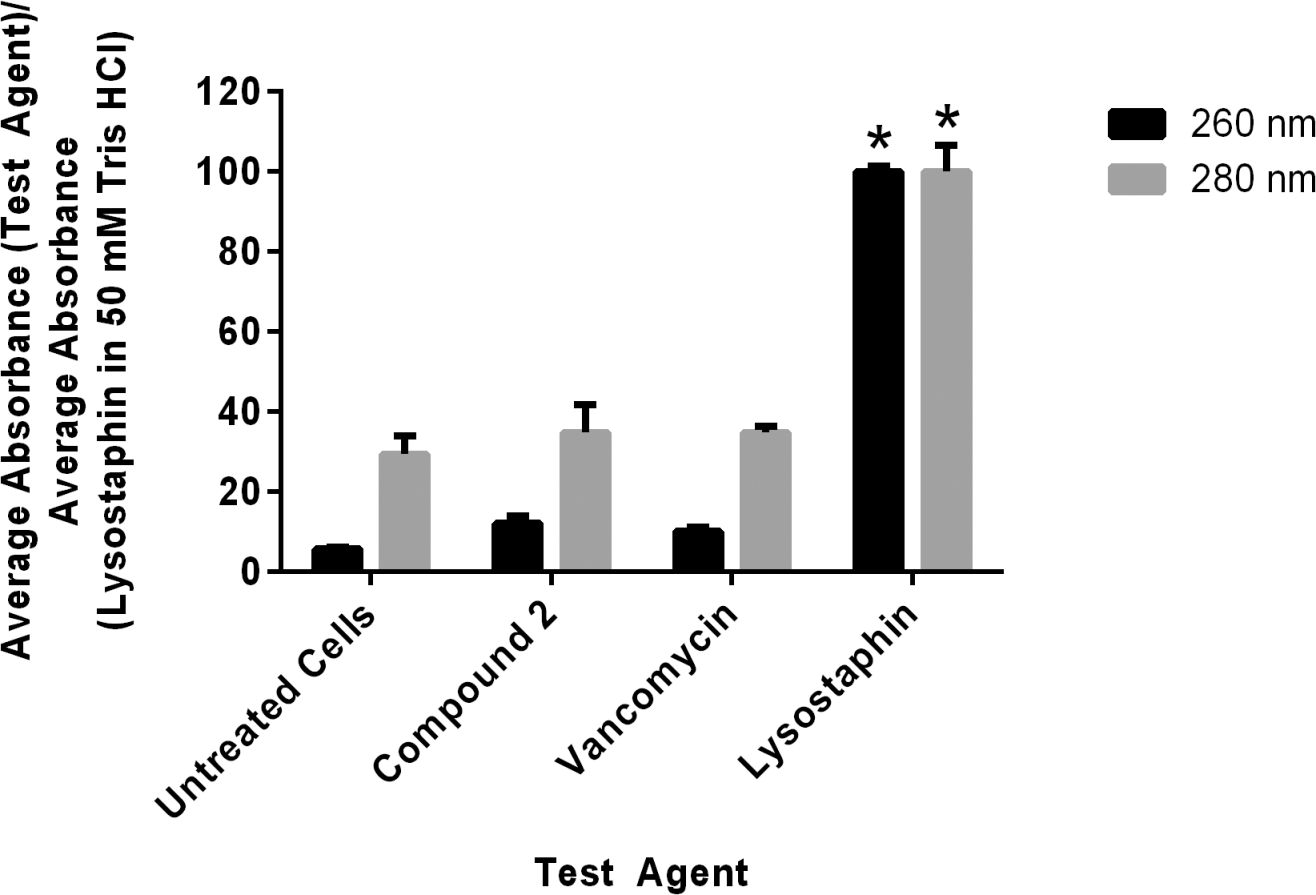
Cell leakage analysis of compound 2, vancomycin, and lysostaphin against methicillin-resistant *Staphylococcus pseudintermedius* SP3. Untreated cells represent the negative control while lysostaphin (in 50 mM Tris-HCl, pH 8.00) served as the positive control. The figure represents the ratio of the average absorbance value obtained for each treatment against the average absorbance value obtained for the positive control (at both 260 and 280 nm). The error bars represent standard deviation values of two experiments where triplicate samples were used for each treatment option. A one-way ANOVA, with post hoc Tukey's multiple comparisons test, *P* ≤ 0.05, demonstrated no statistical difference between the values obtained for compound **2** and vancomycin relative to the untreated cells but significant difference (denoted by the asterisks) in the absorbance values obtained for lysostaphin as compared to both untreated cells and compound **2**.

Disruption of the physical integrity of the bacterial cell membrane (such as formation of pores in the membrane) has been associated with antimicrobials that exhibit rapid bactericidal activity. To assess if the thiazole compounds’ mode of action is disruption of the integrity of the MRSP cell membrane, the leakage of intracellular contents at 260 and 280 nm was analyzed after exposure of bacterial cells to a high concentration of compound **2** (4 × MIC) for 30 minutes. Fig 3 demonstrates that the thiazole compounds do not match the action of lysostaphin (a known membrane-disrupting agent). Less than 20% of the intracellular content (at 260 nm) is released after treatment with the thiazole compound as compared to cells treated with lysostaphin. This result confirms that the thiazole compounds do not act in a manner that involves disruption of the physical integrity of the MRSP cell membrane.

### Toxicity analysis of thiazole compounds

**Fig 4:**
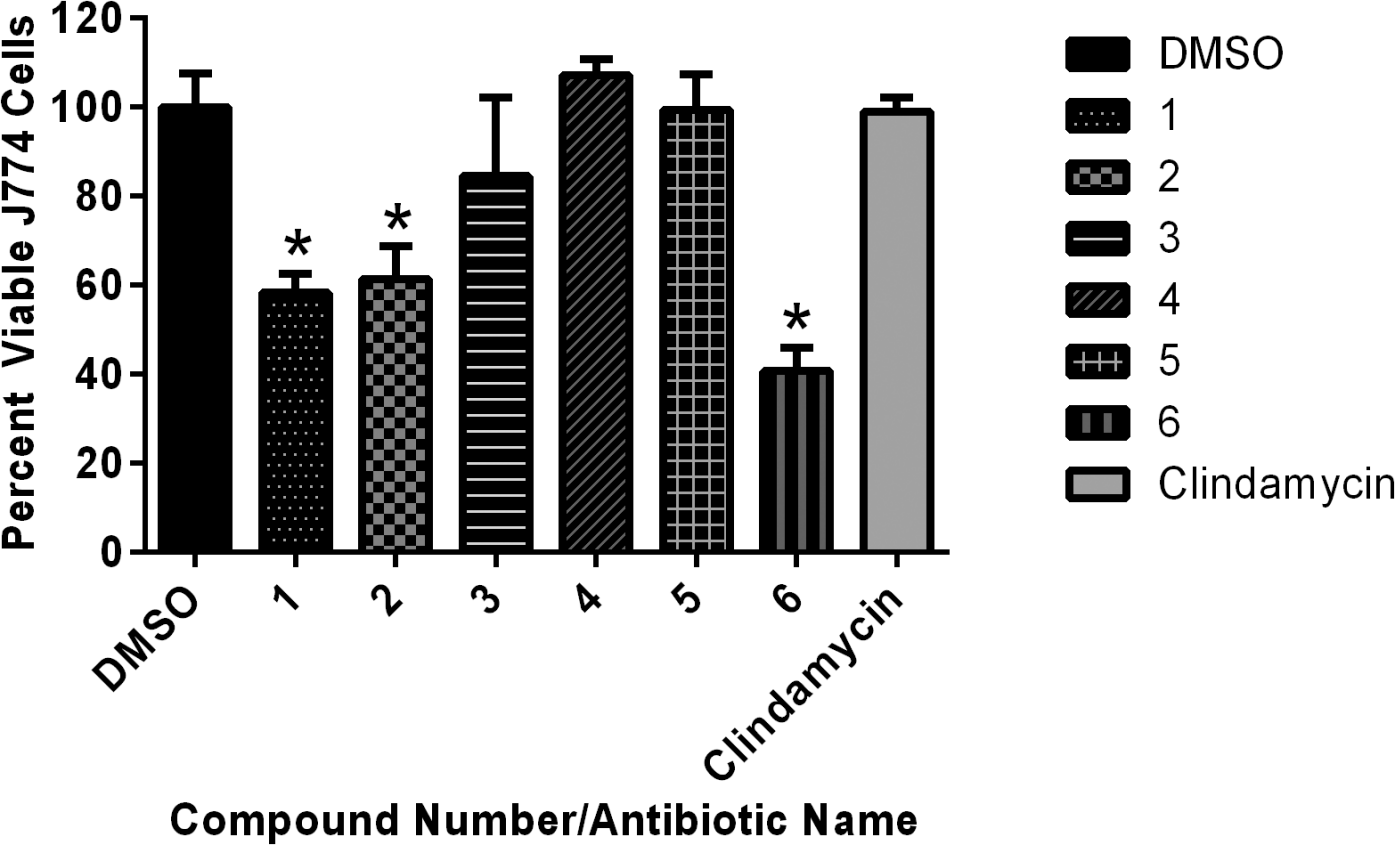
Toxicity analysis of thiazole compounds against murine macrophage (J774.A1) cells. The average absorbance ratio (test agent/DMSO) for cytotoxicity of thiazole compounds at 10 μg/mL against murine macrophage cells (J774.A1) was obtained using the MTS 3-(4,5-dimethylthiazol-2-yl)-5-(3-carboxymethoxyphenyl)-2-(4-sulfophenyl)-2*H*-tetrazolium) assay. DMSO was used as a negative control to determine a baseline measurement for the cytotoxic impact of each compound. The absorbance values represent an average of a minimum of three samples analyzed for each compound. Error bars represent standard deviation values for the corrected absorbance values. A one-way ANOVA, with post hoc Tukey's multiple comparisons test, determined statistical difference between the values obtained for compounds **1**, **2**, and **6** (denoted by the asterisks) relative to the cells treated with DMSO (*P* < 0.05).

Toxicity to host tissues is an important characteristic to assess with new compounds early in the drug discovery process. To determine if the thiazole compounds were toxic to eukaryotic cells, the viability of murine macrophage cells (J774.A1) exposed to each thiazole compound was assessed using the MTS assay. The lead **1** and compounds **2** and **6** proved to be toxic at a concentration of 10 μg/mL (Fig 4). However, derivatives **3**, **4**, and **5** exhibited an improved toxicity profile over the lead compound (matching the results obtained with clindamycin), demonstrating they were not toxic to mammalian cells at 10 μg/mL. This is more than 20-fold higher than the MIC_50_ values obtained for these three compounds against clinical isolates of MRSP.

### Multi-step resistance selection of MRSP to thiazole compounds

**Fig 5:**
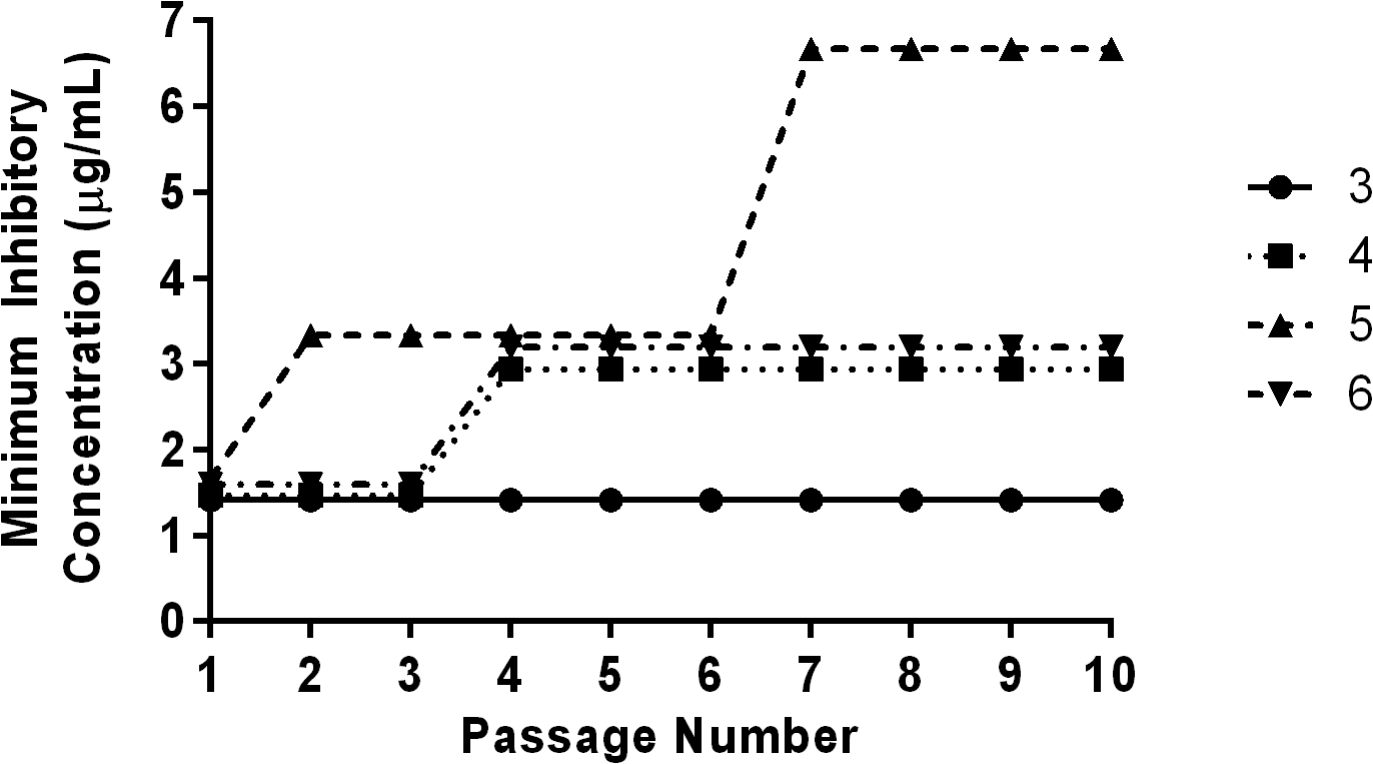
Multi-step resistance selection of thiazole compounds 3–6 against methicillin-resistant *Staphylococcus pseudintermedius*. Bacteria were serially passaged over a ten-day period and the broth microdilution assay was used to determine the minimum inhibitory concentration of each compound against MRSP after each successive passage.

To assess the potential for rapid emergence of resistance of MRSP to the thiazole compounds, a multi-step resistance selection experiment was performed. The initial MICs of compounds **3**, **4**, **5**, and **6** were determined via the broth microdilution method and were found to be 1.41 μg/mL (compound **3**), 1.47 μg/mL (compound **4**), 1.67 μg/mL (compound **5**), and 1.60 μg/mL (compound **6**). Bacteria were then subcultured for ten serial passages to determine if a shift in the MIC of each compound tested would be observed against MRSP. After the second serial passage of compound **5**, there was a two-fold shift in the MIC; the MIC remained stable at 3.34 μg/mL until the seventh passage where a second increase in the MIC was observed to 6.68 μg/mL (Fig 5). Compounds **4** and **5** followed a similar course to one another; the MICs of both compounds remained stable for three passages before a two-fold shift was observed in both compounds after the fourth passage. The MIC did not increase again for both compounds after six additional passages. MRSP was not able to develop resistance to compound **3** even after ten passages.

### Combination therapy and re-sensitization of MRSP to oxacillin in the presence of the thiazole compounds

As MRSP strains exhibit resistance to the effect of β-lactam antibiotics, such as oxacillin, we assessed whether exposure of MRSP to a subinhibitory concentration of the thiazole compounds could re-sensitize the bacteria to the effect of these antibiotics. After initial exposure of MRSP SP3 to a subinhibitory concentration (½ × MIC) of the thiazole compound, the bacteria were next treated with oxacillin. Using the broth microdilution assay, the MIC of oxacillin needed to inhibit MRSP growth in the presence and absence of each thiazole compound was determined. As Table 3 presents, MRSP SP3 was re-sensitized to the effect of oxacillin in the presence of a subinhibitory concentration of all six thiazole compounds. There was a 128-fold reduction in the MIC of oxacillin observed in the presence of ½ × MIC of compounds **1**, **2**, **3**, **4**, and **6** and a 64-fold reduction in the MIC observed with compound **5**. The checkerboard assay was used to assess if these compounds could be used in combination with oxacillin against MRSP. The assay revealed four compounds (**1**, **3**, **5**, and **6**) exhibited a synergistic relationship with oxacillin with ƩFIC values ranging from 0.19 to 0.38.

**Table 3:** Combination testing of thiazole compounds with oxacillin and re-sensitization of methicillin-resistant *Staphylococcus pseudintermedius* SP3 to oxacillin using a subinhibitory concentration (½ × MIC) of thiazole compounds 1–6.

Compound Number	Re-sensitization	ƩFIC^1^
**1**	128-fold	0.19
**2**	128-fold	0.56
**3**	128-fold	0.38
**4**	128-fold	0.63
**5**	64-fold	0.38
**6**	128-fold	0.38

^1^ Results for the FIC index (ƩFIC) are as follows: ≤ 0.5, synergistic (S); > 0.5 to ≤ 4.0, indifference (I); > 4, antagonistic (A).

### Solubility and metabolic stability assessment of compound 3

The solubility of novel compounds in aqueous solutions and stability to host metabolic processes are important to analyze to determine if promising compounds possess suitable drug-like properties. To assess the ability of the thiazole compounds to dissolve in aqueous solutions, a turbidimetric solubility analysis was performed with compound **3** in phosphate-buffered saline. As Table 4 demonstrates, the compound is soluble in PBS up to a concentration of 5.51 μg/mL. This resembles the result obtained for the poorly aqueous drug tamoxifen, indicating compound **3** possesses limited aqueous solubility.

**Table 4:** Evaluation of solubility of thiazole compound 3, reserpine, tamoxifen, and verapamil in phosphate-buffered saline.

Compound Tested	Solubility Limit (μg/mL)^1^
**3**	5.51
Reserpine	19.05
Tamoxifen	5.80
Verapamil	>227.30

^1^ The solubility limit corresponds to the highest concentration of test compound where no precipitate was detected.

To analyze the stability of compound **3** to metabolic processes present in the liver, the compound was incubated with dog liver microsomes. As Table 5 shows, this compound is metabolized very slowly (similar to the drug warfarin) with a NADPH-dependent intrinsic clearance rate of 18.7 μL/min/mg and a half-life of over two hours. The data from Table 5 indicate that this compound is a substrate of a NADPH-dependent metabolic process. There is a nearly three-fold reduction in the intrinsic clearance rate (down to 6.6 μL/min/mg) and a marked increase in the half-life (nearly six hours) of compound **3** in the absence of the co-factor NADPH.

**Table 5:** Evaluation of metabolic stability of thiazole compound 3, verapamil, and warfarin (in duplicate) in dog liver microsomes.

Compound/Drug Tested	NADPH-dependent CL_int_ ^1^(μL/min/mg)	NADPH-dependent T_1/2_ ^2^ (min)	NADPH-free CL_int_ (μL/min/mg)	NADPH-free T_1/2_ (min)
**3**	18.7	123	6.6	351
Verapamil	244	9	0.0	>240
Warfarin	0.0	18.7	0.0	>240

^1^ CL_int_ = microsomal intrinsic clearance rate

^2^ T_1/2_ = half-life

### Post-antibiotic effect of thiazole compounds and antibiotics

*In vitro* pharmacodynamic analysis can provide valuable information regarding establishing a proper dosing regimen for drug candidates. One method to obtain this information is to determine if a compound/drug exhibits a post-antibiotic effect. The PAE for the thiazole compounds, clindamycin, and rifampicin was determined against a clinical isolate of MRSP. Table 6 reveals that all six thiazoles exhibit a long PAE ranging from 8 hours (for compounds **2** and **5**) to > 9 hours (for the remaining four compounds). This is similar to what was observed with rifampicin (PAE > 9 hours) and superior to what was observed with clindamycin (PAE of only two hours).

**Table 6:** *In vitro* post-antibiotic effect (PAE) of thiazole compounds 1–6, clindamycin, and rifampicin against methicillin-resistant *Staphylococcus pseudintermedius* SP3.

Compound Tested	Post-antibiotic Effect (hours)
**1**	>9
**2**	8
**3**	>9
**4**	>9
**5**	8
**6**	>9
Clindamycin	2
Rifampicin	>9

## Discussion

*S*. *pseudintermedius* infections have become a growing problem in veterinary medicine; until fairly recently, the vast majority of infections observed in small animal veterinary facilities could be treated with an array of efficacious antimicrobials [32, 36]. However, the rapid emergence and global spread of multidrug-resistant *S*. *pseudintermedius* (namely MRSP) in the past ten years has presented a significant challenge to veterinary practitioners [3, 37]. Clinical isolates have been identified that exhibit resistance to numerous antibiotic classes, limiting the treatment options available for veterinarians. This underscores the critical need to identify and develop new antibiotics and unique therapeutic strategies to combat this growing medical challenge.

The present study examines the antibacterial potential of novel synthetic thiazole compounds against clinical isolates of MSSP and MRSP. We have previously demonstrated the lead compound and derivative **2** possess potent antimicrobial activity against important strains of multidrug-resistant *S aureus* (primarily MRSA) of concern to both humans and animals [12]. Four additional derivatives (compounds **3**–**6**) of the lead compound were subsequently constructed in an attempt to enhance the antibacterial activity of the lead while mitigating potential toxicity to host tissues. Structural variation constructed focused on modification of the lipophilic alkane side chain of the lead compound, resulting in the butyne analogue **3**, fluorobiphenyl derivative **4**, trifluoromethyl analogue **5**, and the naphthyl derivative **6**. All four were previously found to exhibit potent activity against MRSA so they were also included in this study [23]. As *S*. *pseudintermedius* and *S*. *aureus* share similar genetic and phenotypic characteristics, we hypothesized that these thiazole compounds would possess potent antibacterial activity against MSSP and MRSP. This conjecture was confirmed via the broth microdilution method; all six thiazole compounds exhibited potent antibacterial activity against clinical isolates of both MSSP (MIC_50_ ranged from 0.30–0.80 μg/mL) and MRSP (MIC_50_ ranged from 0.40–1.47 μg/mL). These results proved similar to what was found with clindamycin (MIC_50_ of 0.48 μg/mL against both MSSP and MRSP), an antibiotic of choice for treatment of pyoderma infections [32]. Interestingly, the thiazole compounds retained their antibacterial activity against nine MSSP and MRSP isolates that were found to be resistant to clindamycin and other antibiotics; this indicates there is no cross-resistance present between these antibiotics and the thiazole compounds. This further supports the notion that these thiazole compounds have potential to be used as novel antibacterial agents, particularly against *S*. *pseudintermedius* infections resistant to treatment with other antibiotics.

We were curious to find out if the thiazole compounds possessed bacteriostatic or bactericidal activity. It has been suggested bactericidal antimicrobials have several advantages over their bacteriostatic counterparts, including helping patients recover more rapidly from infection, improving the clinical outcome of disease, reducing the potential emergence of bacterial resistance to the antibiotic, and limiting the spread of infection [38]. Preliminary analysis indicated the thiazole compounds were bactericidal as they possessed MBC_50_ values identical to or two- to three-fold higher than their MIC_50_ values against both MSSP and MRSP isolates. While structural modifications made to the lead thiazole compound did not significantly impact the MIC_50_ and MBC_50_ values found for the subsequently constructed derivatives, there was a significant difference observed in the bacterial killing kinetics against MRSP. A time-kill assay revealed that the alkyne derivative **3** and the naphthyl derivative **6** exhibited superior activity to the lead **1**, rapidly eliminating MRSP within two hours (the lead compound required double the time to achieve the same effect). These results were superior to those obtained with rifampicin, an antibiotic of last resort for pyoderma infections [32].

Rapid bactericidal activity has been shown to be important in the treatment of diseases caused by staphylococci such as endocarditis, meningitis, and osteomyelitis [38, 39]. Thus these thiazole compounds possess a selective advantage over bacteriostatic agents in their ability to be used for treatment of more severe clinical diseases. However, one pitfall of antimicrobials that are rapidly bactericidal is many tend to work as membrane-disrupting agents [38, 40]. Such agents have limited therapeutic applications, almost exclusively being restricted to use as topical ointments [40]. As the thiazole compounds were found to exhibit rapid bactericidal activity, we examined if the mode of action of the thiazole compounds was via disruption of the MRSP cell membrane. A cell leakage analysis confirmed that the thiazole compounds do not physically disrupt the integrity of the bacterial membrane similar to the positive control lysostaphin. The exact mechanism of action of these thiazole compounds against staphylococci is being investigated and will be the subject of a future manuscript.

After confirming the thiazole compounds do in fact possess potent antibacterial activity and are capable of rapidly eliminating MRSP (in a mechanism that does not involve physical disruption of the bacterial cell membrane), we next focused our attention to assessing potential toxicity concerns with these compounds against mammalian cells. Structural modifications made to the lead thiazole compound played an important role in enhancing the toxicity profile of the thiazoles. The lead compound and biphenyl derivative **2** were found to be toxic to murine macrophage cells at a concentration of 10 μg/mL. Surprisingly, replacement of the alkyl moiety in the lead with an alkyne, monofluoro, or trifluoromethyl group (as in compounds **3**–**5**, respectively) significantly improved the toxicity profiles of the compounds. These three derivatives were not toxic to murine macrophage cells at 10 μg/mL which represents a greater than 20-fold difference over the MIC_50_ values determined against MRSP.

The ability of bacteria to develop resistance rapidly to antimicrobial compounds is important to assess early in drug discovery. Previously, we have reported results of a single-step resistance selection experiment that demonstrated MRSA is unlikely to develop rapid resistance to thiazole compounds **1** and **2** [13]. We decided to extend this analysis to the newest thiazole derivatives (compounds **3**–**6**) against MRSP but with an additional twist–testing if bacterial resistance could be induced after repeated exposure to each compound over 10 serial passages. There was no change observed in the MIC for compound **3**, a two-fold increase in the MIC for compounds **4** and **5**, and a four-fold increase in the MIC of compound **6** after 10 passages. Collectively, the results provide data supporting a low probability of MRSP-resistance developing rapidly to these thiazole compounds (as a greater than four-fold increase, as compared to the initial MIC, was not observed for any of the compounds tested).

While discovery of novel antimicrobials for use in monotherapy is one important avenue to address the burden of multidrug-resistant bacterial infections, other therapeutic strategies must be explored. Recently, suppression of MRSA resistance to β-lactam antibiotics by using these agents in combination with other antimicrobial compounds has been explored as an alternative therapeutic strategy [41, 42]. This has the potential to prolong the usage of β-lactam antibiotics (particularly those that are less susceptible to degradation by β-lactamase such as first-generation cephalosporins) in the clinical setting. As first-generation cephalosporins are frequently used as first-line agents to treat staphylococcal infections present in small animal veterinary practices, β-lactam antibiotics still play a very integral role in the clinic [43]. Prolonging the ability to use these antibiotics against resistant strains of staphylococci, such as MRSP, is extremely important. No studies have been reported thus far testing the ability of antimicrobial compounds to suppress MRSP resistance to β-lactam antibiotics. In an earlier study, we demonstrated that thiazole compound **2** can re-sensitize vancomycin-resistant *Staphylococcus aureus* (VRSA) to the effect of vancomycin [13]. As glycopeptide antibiotics (such as vancomycin) and β-lactam antibiotics both target cell wall synthesis in bacteria, we hypothesized that the thiazole compounds would be able to re-sensitize MRSP to the effect of β-lactam antibiotics. Bacterial susceptibility to oxacillin is used as a standard to determine if bacteria are sensitive or resistant to β-lactam antibiotics (as resistant strains can appear sensitive to other β-lactam antibiotics *in vitro* but exhibit resistance to these antibiotics *in vivo*) [43]. Using a clinical isolate identified as MRSP, we used the broth microdilution assay to first confirm that the isolate was resistant to oxacillin (MIC = 128 μg/mL). Next, the isolate was exposed to a subinhibitory concentration (½ × MIC) of each thiazole compound for 30 minutes; afterward, the broth microdilution assay was used to determine the sensitivity of the isolate to oxacillin. All six compounds demonstrated the ability to re-sensitize MRSP to oxacillin (a 64 to 128-fold reduction in the MIC of oxacillin was observed after pre-treatment with the thiazole compounds). Furthermore, the checkerboard assay confirmed that compounds **1**, **3**, **5**, and **6** exhibited a synergistic relationship with oxacillin with ƩFIC values ranging from 0.19 to 0.38. This analysis confirmed that in addition to being used as antimicrobial agents alone in the treatment of *S*. *pseudintermedius* infections, the thiazole compounds have the potential to be used i.) in combination with β-lactam antibiotics against MRSP or ii.) to suppress resistance of MRSP to β-lactam antibiotics. This expands the potential therapeutic applications of these compounds beyond just use in monotherapy. Additionally, the finding that thiazole compounds can be effectively combined with oxacillin, an inhibitor of peptidoglycan biosynthesis, against MRSP paves the way for further investigation of combination therapy of thiazole compounds with other cell wall synthesis inhibitors.

After confirming the thiazole compounds have potential use as antibacterial agents for the treatment of *S*. *pseudintermedius* infections, it was important to assess if the newly constructed derivatives exhibited suitable drug-like properties (such as aqueous solubility for drug absorption and metabolic stability). As compound **3** appeared the most promising drug candidate (due to its rapid bactericidal activity, improved toxicity profile, low induction of MRSP resistance, and ability to suppress MRSP resistance to β-lactam antibiotics), it was selected for further analysis. Previously, it was found that the lead compound has moderate aqueous solubility in phosphate-buffered saline (solubility limit of 20.56 μg/mL) [12]. Substitution of the alkane in the lead compound with an alkyne (as in compound **3**) resulted in a reduction in the aqueous solubility observed. However, this substitution significantly enhanced the metabolic stability of compound **3**, when compared to the lead compound. Previously the lead compound was cleared by human liver microsomes at a rate of 80.3 μL/min/mg and had a half-life just under 30 minutes [12]. When the same analysis in human liver microsomes was performed for the modified derivative **3**, a significant improvement in the metabolic stability profile of this compound was observed (clearance rate decreased to 3.7 μL/min/mg and half-life was more than 240 minutes) [23]. In this study, compound **3** was analyzed using dog liver microsomes (to compare if the results found in human liver microsomes could be confirmed, given that metabolic processes in dogs and humans differ). Compound **3** was found to have a metabolic clearance rate of 18.7 μL/min/mg (greater than four-fold improvement in how rapidly the compound is metabolized and cleared from liver cells compared to the lead compound) in dog liver microsomes. Additionally, the half-life of 123 minutes for compound **3** is a significant improvement over the result found for the lead compound. This result is important as it ensures this compound is unlikely to be rapidly metabolized and excreted from the patient’s body, thus decreasing the size and frequency of doses needed to be administered to treat a patient afflicted with a bacterial infection attributed to *S*. *pseudintermedius*. Though compound **3** possesses poor aqueous solubility, formulation technology has been shown to be an effective strategy to employ to overcome this limitation and advance promising compounds to the market [44]. Identification of this limitation early in the drug discovery process provides an area for formulation scientists and medicinal chemists to address to propel compound **3** into further drug discovery stages.

The metabolic stability analysis performed with compound **3** provided valuable evidence that fewer doses of this compound would need to be administered to treat a patient dealing with an infection. The post-antibiotic effect analysis performed further validated this observation. PAE analysis has been shown to be an important parameter to establish an optimal dosing regimen (size and frequency of doses given to patients) [45]. As compound **3** exhibits a long PAE (> 9 hours) against MRSP, this indicates bacteria are very slow to recover after exposure to this compound. Thus, patients would need to be subjected to fewer doses of this particular compound (as compared to clindamycin, for example, where the PAE against MRSP was found to be only two hours). This is clinically significant as antimicrobials that demonstrate a PAE (in particular an extended PAE as is observed with the thiazole compounds) possess several advantages including reduced costs (fewer doses needed for treatment), limited toxicity to host tissues, and greater patient cooperation in sticking to the prescribed treatment regimen [46].

## Conclusion

In this study we have demonstrated novel thiazole compounds synthesized by our research group do in fact possess potent antibacterial activity against clinical isolates of both methicillin-sensitive and methicillin-resistant *S*. *pseudintermedius*. The lead compound and five derivatives are capable of inhibiting bacterial growth at concentrations similar to clindamycin, a drug of choice in canine pyoderma infections. Though all six compounds are bactericidal, two derivatives (**3** and **6**) exhibit superior killing kinetics by completely eliminating MRSP within two hours (were superior to rifampicin). Compound **3** appears to be the most suitable derivative to continue with further studies involving *S*. *pseudintermedius* as it is not toxic to mammalian cells at a concentration 20-fold higher than its MIC_50_ value against MRSP. Additionally, MRSP is predicted not to develop rapid resistance to this compound even after multiple exposures/doses. Furthermore, this compound exhibits a markedly improved metabolic stability profile compared to the lead compound. While the thiazole compounds show promise for use alone to treat *S*. *pseudintermedius* infections, these compounds also demonstrate the ability to re-sensitize MRSP to the effect of oxacillin; this opens the door for the potential use of these compounds to prolong the utility of β-lactam antibiotics for treatment of infections caused by MRSP.
